# Unrecognized bilateral temporomandibular joint dislocation after general anesthesia with a delay in diagnosis and management: a case report

**DOI:** 10.1186/1752-1947-7-243

**Published:** 2013-10-18

**Authors:** Suri Pillai, Mojca Remskar Konia

**Affiliations:** 1 Department of Anesthesiology, University of Minnesota, B-515 Mayo Memorial Building, 420 Delaware Street SE, Mayo Mail Code 294, Minneapolis, MN 55455, USA

## Abstract

**Introduction:**

Anterior bilateral temporomandibular joint dislocation is not an uncommon occurrence and has been reported before. However, its diagnosis can easily be overlooked, especially by clinicians who are unfamiliar with this pathology. Continuous discussion of the pathology is required to prevent delays in diagnosis, which can lead to long-term sequelae for the patient.

**Case presentation:**

We present the case of a 66-year-old Somali woman who experienced a bilateral anterior temporomandibular joint dislocation after a general anesthetic for an exploratory laparotomy for excision of a pelvic sarcoma. She first presented in the intensive care unit with preauricular pain and an inability to close her mouth, and was initially misdiagnosed and treated for a muscle spasm. The cause of her misdiagnosis was multifactorial - opioid-related sedation, language and cultural barrier, and unfamiliarity with the pathology. Her diagnosis was proven 18 hours after the completion of surgery with a plain X-ray. A manual closed reduction was performed with minimal sedation by oral surgery.

**Conclusion:**

We provided an in-depth discussion of temporomandibular joint dislocation and suggest a simple test that would prevent delayed diagnosis of temporomandibular joint dislocation in any patient undergoing general anesthesia. A normal mandibular excursion should be tested in every patient after surgery in the postoperative care unit, by asking the patient to open and close their mouth during the immediate postoperative recovery period or passively performing the range of motion test.

## Introduction

Temporomandibular joint (TMJ) evaluation is a component of the preoperative airway physical examination as suggested by The American Society of Anesthesiologist practice guidelines for the management of the difficult airway [1]. The majority of anesthesiologists focus on the TMJ function evaluation as it relates to optimal intubating conditions. However, TMJ dysfunction or dislocation caused by an anesthesiologist’s manipulation is also possible. The incidence of TMJ dysfunction following endotracheal intubation is reported to be 5% [2]. Furthermore, TMJ injuries represent 10% (27 out of 266) of airway trauma claims in closed claims analysis, 16 being TMJ pain and 11 being TMJ dislocation [3]. TMJ dislocation is not always obvious and can be easily overlooked if special attention is not paid to the evaluation of the TMJ perioperatively. Failure to recognize TMJ dislocation can lead to permanent damage and a need for surgical intervention [4].

We describe the delayed diagnosis and treatment of bilateral TMJ dislocation in a 66-year-old woman following an otherwise uneventful general anesthetic. Factors that led to the delayed diagnosis and actions that could have prevented it are discussed. Written and verbal consent from the patient has been obtained.

## Case presentation

Our patient was a 66-year-old Somali non-English-speaking woman, weighing 65.2kg with a height of 1.57m. She had a past medical history of *Helicobacter pylori* infection, acid reflux disease, hyperlipidemia and diabetes mellitus type II. She was recently diagnosed with a large pelvic sarcoma that required surgical excision.

A preoperative evaluation was unremarkable. Her Mallampati score was 2 and her cervical, atlanto-axial and atlanto-occipital joints demonstrated full range of motion. She had a normal mouth opening measuring three fingerbreadths. She was noted to have a somewhat hypoplastic mandible and her thyromental distance was measured to be less than three fingerbreadths. She was premedicated with 2mg of midazolam then taken to the operating room, where she underwent a standard intravenous induction with 50μg of fentanyl, 80mg of lidocaine, 150mg of propofol and 50mg of rocuronium. She was reported to be easy to mask without an oral airway.

A direct laryngoscopy was performed once with a Miller 2 blade; a grade 1 view was obtained and intubation was atraumatic. Following intubation, a nasogastric tube was placed without difficulty. She underwent placement of bilateral ureteral stents and a large pelvic sarcoma excision over the course of 11 hours. Spontaneous respirations returned by the end of the procedure; however, she was slow to respond to commands so she was brought to the post-operative care unit (PACU) on a T-piece and supplemental oxygen. She was extubated without difficulty and transferred to the surgical intensive care unit (SICU) in the evening.

**Figure 1:**
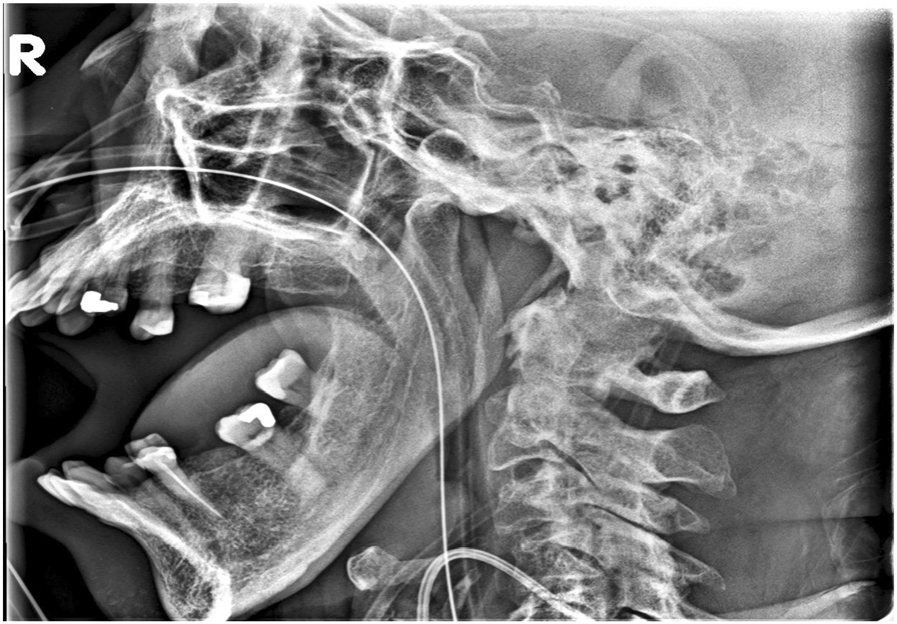
Lateral X-ray depicting anterior dislocation of the temporomandibular joint.

During the night, a nurse notified the on-call surgery resident that our patient was unable to close her mouth, and appeared to be in severe pain. After examining our patient, a diagnosis of muscle spasm was made and a hydromorphone patient-controlled analgesia was ordered. In the morning, the primary surgical team, followed by the SICU team, examined our patient, who was still unable to close her mouth. Both teams considered the diagnosis of muscle spasm as the most likely etiology. A rotating anesthesiology resident on the SICU team proposed a diagnosis of TMJ dislocation. Imaging confirmed the diagnosis of anterior bilateral TMJ dislocation (Figure 1). The oral surgery team were consulted and our patient was seen in the afternoon. With 1mg of diazepam, the dislocation was reduced without difficulty via an intraoral closed reduction technique.

It is uncertain the exact time at which the dislocation occurred in our patient, but it should be noted that there was at least an 18-hour window from the time the surgery resident was notified of the patient’s inability to close her mouth to the time of the reduction. Unfortunately for our patient, the pathology went undiagnosed due to unfamiliarity of the surgical and SICU teams with the condition.

## Discussion

In our patient, the diagnosis and management of TMJ dislocation was delayed. It is unclear when the dislocation occurred; it could have been any time during direct laryngoscopy and intubation or extubation, or anytime in the postoperative period [5-11]. Several factors were present that compounded the delay in diagnosis: slow recovery and extubation of our patient in the PACU; the language barrier; generous pain medication administration in the PACU and SICU, which made the patient quite somnolent; and unfamiliarity of the surgical and SICU teams with the possibility of TMJ dislocation during manipulation of the jaw under anesthesia.

Anterior TMJ dislocation is the most prevalent dislocation described from non-traumatic causes. It occurs when the mandibular condyle(s) travels anterior to the articular eminence. Anatomic and physiologic characteristics of the joint, excessive movements, and interruption of the normal sequence of muscle activation are all considered contributing factors to the development of anterior TMJ dislocation [12]. Besides dislocation, pathologies such as anterior dislocation of TMJ meniscus, with or without reduction, and myofacial pain dysfunction syndrome should be considered in the differential diagnosis [12,13]. Several studies have reported post-intubation TMJ dysfunction presenting as changed range of motion and/or periauricular pain in approximately 5% of cases [2,12]. Factors related to TMJ dysfunction after endotracheal intubation have been studied by Martin *et al.,* and were shown to be female gender, interincisal distance, previous TMJ pain with or without headache, and age [14]. Of note, Mallampati score and duration of intubation were not risk factors for TMJ dysfunction.

TMJ dislocation represents 3% of all dislocations throughout the body [15]. Anterior TMJ dislocation has been described during maneuvers where there is excessive opening of the mouth, such as yawning, eating, dental procedures, transesophageal echo probe placement, orotracheal intubation via direct laryngoscopy, and laryngeal mask airway placement [5-11,16-18].

Clinical presentation of anterior TMJ dislocation after anesthesia can vary significantly. Some patients present with severe pain in the periauricular area and an inability to close the mouth, which quickly suggest the diagnosis [19]. In other cases, however, the presentation can be subacute, mouth opening less obvious and pain less impressive, so the diagnosis can be easily missed [13,20]. Delay of diagnosis for over a month has been reported in patients with a decreased level of consciousness due to a variety of factors such as head trauma or sedation after cardiac surgery [13,20,21]. In some patients it is not until muscle spasm develops several hours later that the patient begins complaining of pain. It should be noted that spasms of the mastication muscles alone would be unlikely to keep the mouth open in a fixed and locked position unless there was an associated dislocation, because the muscles that close the mouth are much more powerful than the muscles that open the mouth.

## Conclusion

A conscious patient with TMJ most often presents with an inability to close the mouth, salivation, impaired speech and a depression that is palpable in the preauricular area. In addition, the patient usually communicates significant pain and anxiety. The diagnosis is mainly clinical, and if there is no history of trauma to the face, a reduction procedure may be performed without imaging or oral surgery consultation [22]. Several approaches to closed reduction have been described and are reviewed in an article by Chan *et al.*[23]. Light procedural sedation is usually quite sufficient for allowing a successful manual reduction. Various benzodiazepines, narcotics and induction agents, such as propofol, have all been described at various doses. Diagnosis and treatment of TMJ dislocation should occur promptly. After dislocation occurs, spasms of the masseter and pterygoid muscles may worsen over time, causing the mandible to contract into the dislocated position, therefore making the reduction procedure more difficult [24]. If left untreated for longer than 14 days, fibrosis and even fractures become increasingly apparent [13,25].

Following successful reduction of anterior TMJ dislocation the patient should be advised to limit extreme mouth opening and to employ a soft mechanical diet for several days. A stabilization brace that wraps under the mandible and over the cranium while at rest may be beneficial in preventing re-dislocation in the immediate post-reduction period. Over-the-counter non-steroidal anti-inflammatory drugs are usually sufficient for pain control. Oral surgery consultation is warranted in cases of failed reduction attempts, associated fractures, or in patients with a history of repeated chronic dislocation, and operative intervention may be warranted [23].

We would like to emphasize the importance of thorough perioperative evaluation of the TMJ. During preoperative evaluation, the focus should be on determining any limitations in the range of motion of the TMJ and the ability to voluntarily protrude the mandible as suggested by the American Society of Anesthesiology guidelines. Furthermore, history of previous injury to the jaw, any periauricular pain, with or without headache, and any clicking or grating sounds during mandibular motion should be noted. Any of these signs and symptoms may indicate existing TMJ dysfunction and significantly increase the likelihood of increased severity of postoperative symptoms. Agro, *et al.* reported increased symptoms following endotracheal intubation in 44% of patients with preexisting TMJ dysfunction [2]. It would seem prudent to discuss the possibility of TMJ dysfunction with any patient undergoing general anesthesia. Furthermore, to exclude TMJ dislocation or dysfunction, we suggest a simple test of normal mandibular excursion by asking every patient undergoing a general anesthetic to open and close their mouth during the immediate postoperative recovery period or by checking the passive range of motion of the TMJ joint.

## Consent

Written informed consent was obtained from the patient for the publication of the case report and the accompanying image. A copy of the written consent is available for review by the Editor-in-Chief of this journal.

## Abbreviations

PACU: Postoperative care unit; SICU: Surgical intensive care unit; TMJ: Temporomandibular joint.

## Competing interests

The authors declare that they have no competing interests.

## Authors’ contributions

SP was the primary contributor to manuscript preparation, editing, design and acquisition of data. MRK critically revised the article for relevant intellectual content and contributed to intellectual references. Both authors read and approved the final manuscript.
